# Queries Addressed to Mr. Smerdon

**Published:** 1815-03

**Authors:** 


*For the Medical and Physical Journal.*

### *Queries addressed to Mr. Smerdon.*

ONE of the most eminent surgeons in this metropolis for a scientific knowledge of his profession, in his investigation of the proximate cause of inflammation, observes, that he shall not be expected to examine those causes designated under the terms archeus, anima, living principle, &c. as their existence must be first demonstrated before any interpretation founded on their agency can be admitted. I should, therefore, feel much obliged to Mr. Smerdon, if he would have the goodness to inform me, what are his proofs, that irritability is independant of living organization; I should also thank him for the evidence of the existence of a nervous fluid, and its emanation from the brain; and to know whether he uses the term theory as synonimous with hypothesis.

*Jan. 25,* 1815. LONDINENSIS.

